# Exploring the causality of appendectomy and ischaemic heart disease: a Mendelian randomization study and meta-analysis

**DOI:** 10.3389/fcvm.2024.1443906

**Published:** 2024-08-06

**Authors:** Shuai Wang, Tao Zhang, Yuanlin Sun, Yiwei Yao, Dongliang Yang, Xueyuan Cao

**Affiliations:** Department of Gastric and Colorectal Surgery, General Surgery Center, The First Hospital of Jilin University, Changchun, Jilin, China

**Keywords:** Mendelian randomization, appendectomy, ischaemic heart disease, myocardial infarction, angina pectoris

## Abstract

**Background:**

The risk of ischaemic heart disease (IHD) is increased in appendectomy patients, but it is not clear whether there is a causal relationship. We aimed to systematically estimate the causal relationship between appendectomy and IHD and its subtypes, acute myocardial infarction (AMI) and angina pectoris (AP), using Mendelian randomization (MR) study methods and meta-analysis.

**Methods:**

As the discovery cohort analysis, we extracted independent genetic variants strongly associated with appendectomy from the FinnGen study (28,601 cases) as instrumental variables (IVs). Genome-wide association study (GWAS) from UK Biobank were selected for outcome data. A first two-sample MR analysis was then conducted. As the replication cohort, IVs associated with appendectomy were extracted in the UK Biobank (50,105 cases). GWAS from the FinnGen study were selected for outcome data. A second MR analysis was then performed. Finally, meta-analyses were applied to assess the combined causal effects of the MR results.

**Results:**

In the discovery cohort, there was a significant positive causal relationship between appendectomy and IHD and its subtypes AMI and AP. The replication cohort only found a positive causal relationship between appendectomy and AMI. Meta-analysis showed a positive causal relationship between appendectomy and IHD (OR: 1.128, 95% CI: 1.067–1.193, *P *= 2.459e-05), AMI (OR: 1.195, 95% CI: 1.095–1.305, *P *= 6.898e-05), and AP (OR: 1.087, 95% CI: 1.016–1.164, *P *= 1.598e-02).

**Conclusions:**

This comprehensive MR analysis suggests that genetically predicted appendectomy may be a risk factor for the development of IHD and its subtypes AMI and AP. We need to continue to pay attention to these links.

## 1 Introduction

Acute appendicitis is a prevalent cause of acute abdominal pain on a global scale. The estimated lifetime risk of developing this condition is approximately 7%–8% (1). Appendectomy stands as the conventional treatment for appendiceal conditions, including acute appendicitis (2). Recent research has increasingly delved into the health implications of appendectomy on patients. Some studies have suggested that appendectomy could serve as a predisposing factor for conditions such as colorectal cancer (3), gallstones (4), and Crohn's disease (5). Conversely, evidence has indicated that appendectomy may act as a protective factor against ulcerative colitis (5), Parkinson's disease (6), and amyotrophic lateral sclerosis (6), among others. The majority of these studies have concentrated on establishing a causal link between appendectomy and gastrointestinal disorders.

In contrast, two studies have focused on the relationship between appendectomy and the risk of IHD. IHD has been considered the top cause of mortality globally (7). IHD is recognized as a leading cause of mortality on a global scale. The prevalence, incidence, and fatality rates of IHD have shown a rising trend worldwide from 1990 to 2019, posing a significant public health concern (8, 9). A large cohort study from a Swedish population showed that appendectomy before 20 years of age was associated with acute myocardial infarction (HR: 1.33, 95% CI: 1.05–1.70) (10). Similarly, another cohort study from Taiwan, China, showed that appendectomy was strongly associated with an increased risk of IHD within three years (HR: 1.54, 95% CI: 1.29–1.84) (11). Nevertheless, accurately assessing the risk of IHD and AMI in individuals who have undergone appendectomy is challenging due to the presence of confounding variables in conventional epidemiological investigations.

MR is a novel epidemiological analysis technique that leverages genetic variability to evaluate causal associations between exposures and clinical outcomes (12, 13). The method is based on the principle of random distribution in biology, making its results immune to potential confounders and reverse causation. The MR approach can provide strong evidence to support the causal role of risk factors on outcomes because the distribution of genetic variation is randomized across generations. Single nucleotide polymorphism (SNP) is a kind of genetic variation known to be valuable. SNPs were chosen as an instrumental variable in this study. This study systematically assessed the causal relationship between appendectomy and IHD and its two main subtypes, AMI and AP, using MR methods and meta-analysis. The findings can contribute valuable insights into the screening and diagnosis of IHD in patients who have undergone appendectomy.

## 2 Methods

### 2.1 Research design

**Figure 1:**
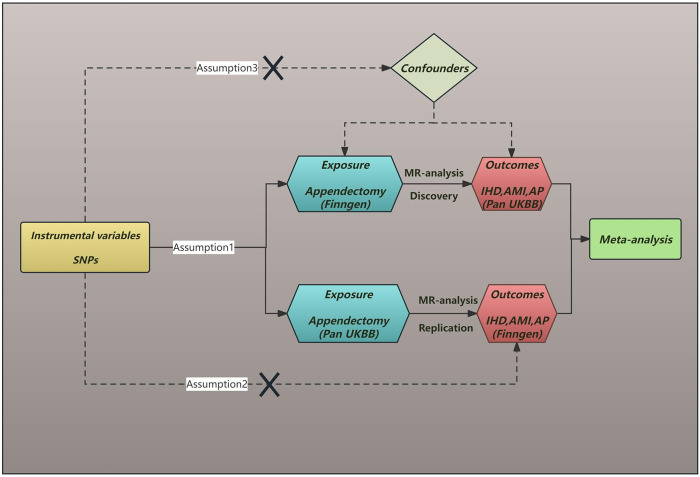
The diagram of Mendelian randomization assumption. SNPs, single nucleotide polymorphisms; IHD, ischaemic heart disease; AMI, acute myocardial infarction; AP, angina pectoris.

In MR research, IVs must meet three basic requirements: (1) IVs must be directly related to exposure factors. (2) IVs are not associated with confounders that may affect the relationship between exposure and outcome. (3) IVs do not influence outcomes other than the exposure pathways that influence outcomes (Figure 1) (14). There was no need to get informed consent or ethical approval for this study again because all of the data were taken from published sources, and the informed consent and approval were received. This study was conducted according to the STROBE-MR guidelines (Supplementary Table S1).

### 2.2 Exposure data acquisition

GWAS studies of appendectomy (28,601 cases and 383,580 controls) in the discovery cohort were obtained from the FinnGen study. The FinnGen study is a large-scale genomics initiative that has analyzed over 500,000 Finnish biobank samples and correlated genetic variation with health data to understand disease mechanisms and predispositions. The project is a collaboration between research organisations and biobanks within Finland and international industry partners. GWAS summary statistics can be downloaded from the FinnGen study (https://www.finngen.fi/en) (15). Detailed information is provided in Table 1 and Supplementary Table S6.

**Table 1:** Information of genome-wide association summary data.

Characteristic	Resource	Sample size	Population
Appendectomy (discovery cohort)	FinnGen (R10)	28,601 cases and 383,580 controls	European
IHD (discovery cohort)	Pan UKBB	37,672 cases and 382,052 controls	European
AMI (discovery cohort)	Pan UKBB	10,141 cases and 410,390 controls	European
AP (discovery cohort)	Pan UKBB	21,286 cases and 399,245 controls	European
Appendectomy (replication cohort)	Pan UKBB	50,105 cases and 370,368 controls	European
IHD (replication cohort)	FinnGen (R10)	69,008 cases and 342,173 controls	European
AMI (replication cohort)	FinnGen (R10)	26,060 cases and 343,079 controls	European
AP (replication cohort)	FinnGen (R10)	36,875 cases and 343,079 controls	European

IHD, ischaemic heart disease; AMI, acute myocardial infarction; AP, angina pectoris.

The GWAS studies of appendectomy (50,105 cases and 370,368 controls) in the replication cohort were obtained from the UK Biobank studies. The UK Biobank study is an ongoing cohort study initiated by recruiting about 500,000 adults between 2006 and 2010. It is a large-scale open database with hundreds of thousands of individuals' genotype data paired with electronic health records and survey measures. GWAS summary statistics can be downloaded from the UK Biobank (https://pan.ukbb.broadinstitute.org/) (16). Detailed information is provided in Table 1 and Supplementary Table S6.

### 2.3 Outcome data acquisition

In the discovery cohort, data for IHD (37,672 cases and 382,052 controls), AMI (10,141 cases and 410,390 controls) and AP (21,286 cases and 399,245 controls) were all obtained from the latest GWAS data publicly available from the UK Biobank.

In the replication cohort, data for IHD (69,008 cases and 342,173 controls), AMI (26,060 cases and 343,079 controls) and AP (36,875 cases and 343,079 controls) were similarly obtained from the latest GWAS data publicly available from the FinnGen study.

Detailed information is provided in Table 1 and Supplementary Table S6.

### 2.4 Selection of instrumental variables

For the discovery cohort, in constructing IVs, genome-wide SNPs with *P* < 5e-08 were extracted from the GWAS pooled data, but not enough SNPs were obtained, so the criterion of *P* < 5e-06 was chosen to obtain a sufficient number of IVs, and those with a longer physical distance (≥ 10,000 kb) and less possibility of linkage disequilibrium (R^2^ < 0.001) were retained. We queried the possible phenotypes for each SNP associated with IHD, AMI, and AP by LDtrait (https://ldlink.nci.nih.gov/?tab=ldtrait) (17) and SNPs commonly recognized confounding factors related to IHD, AMI, and AP were removed, such as type 2 diabetes mellitus (18) and waist circumference adjusted for body mass index (Supplementary Table S2) (19). To avoid weak instrumental variable bias, we evaluated the SNP-exposure association strengths using the F = BETA^2^/SE^2^ (Supplementary Table S3) for each SNP (20, 21). When the SNPs had an F value > 10, we considered a strong association between the selected IVs and exposure. We also excluded SNPs with a minor allele frequency ≤0.01, and removed palindromic sequences in IVs. Finally, we removed outliers using the MR-PRESSO test before each MR analysis.

For the replication cohort, genome-wide significant SNPs (*P* < 5e - 08) were extracted from the GWAS pooled data, and then eligible SNPs were screened by the same screening method (Supplementary Table S4).

### 2.5 MR analysis

The MR analyses in this study were performed in R 4.2.1 software. “Two Sample MR” and “MR-PRESSO” in R were used. The inverse variance weighting (IVW) model is the most powerful method for detecting causality in two-sample MR analysis (22). This study used the IVW method as the most dominant method for calculating causal effects. IVW method estimates the causal effect of exposure on outcome by combining the ratio estimates for each SNP, which essentially transforms the MR estimate into a weighted regression of the SNP outcome effect on the SNP exposure effect (13). The MR-Egger methodology relaxes the requirement of no level of multicollinearity between SNPs. Instead, it assumes no correlation between gene exposure associations and the direct effect of genetic variation on outcomes. This is the inside assumption (Instrumental Strength Independent of Direct Effect) and is a weaker requirement than the more stringent exclusion restriction criterion. A disadvantage of the MR-Egger method is that it tends to have low statistical power and is particularly susceptible to weak instrumental bias (12). The weighted median method provides unbiased estimates even if up to 50% of the information derives from invalid IVs. The weighted median method is more accurate than MR-Egger but does not address selection bias (23). Therefore, the Mg-Egger method and the weighted median method are complementary to the IVW method as preliminary sensitivity analyses of the results.

Cochran's Q-test assessed the heterogeneity of the IVW model. Cochran's Q-test of *p *< 0.05 indicates heterogeneity (24). If there is no heterogeneity, we use a fixed effects model. Otherwise, a random effects model is used (25). MR-Egger intercept test was performed to assess whether the included SNPs were potentially horizontally pleiotropic, and a *p*-value of <0.05 indicated the presence of pleiotropy (26). The leave-one-out sensitivity test eliminates SNPs to determine the sensitivity of individual SNPs in this MR study. This study also used scatter, forest, and funnel plots for visualization and analysis (27). Finally, to present a comprehensive and accurate picture of the causal relationship between appendectomy and IHD, AMI, and AP, we used meta-analyses to assess the combined causal effects of MR outcomes. *P* < 0.05 was considered statistically significant (two-sided). We used the odds ratio (OR) and 95% confidence interval (CI) to assess the relative risk between appendectomy and IHD, AMI, and AP. The statistical power was calculated by the mRnd website (https://shiny.cnsgenomics.com/mRnd/) (Supplementary Table S5) (28).

## 3 Results

### 3.1 Results of the discovery cohort

**Figure 2:**
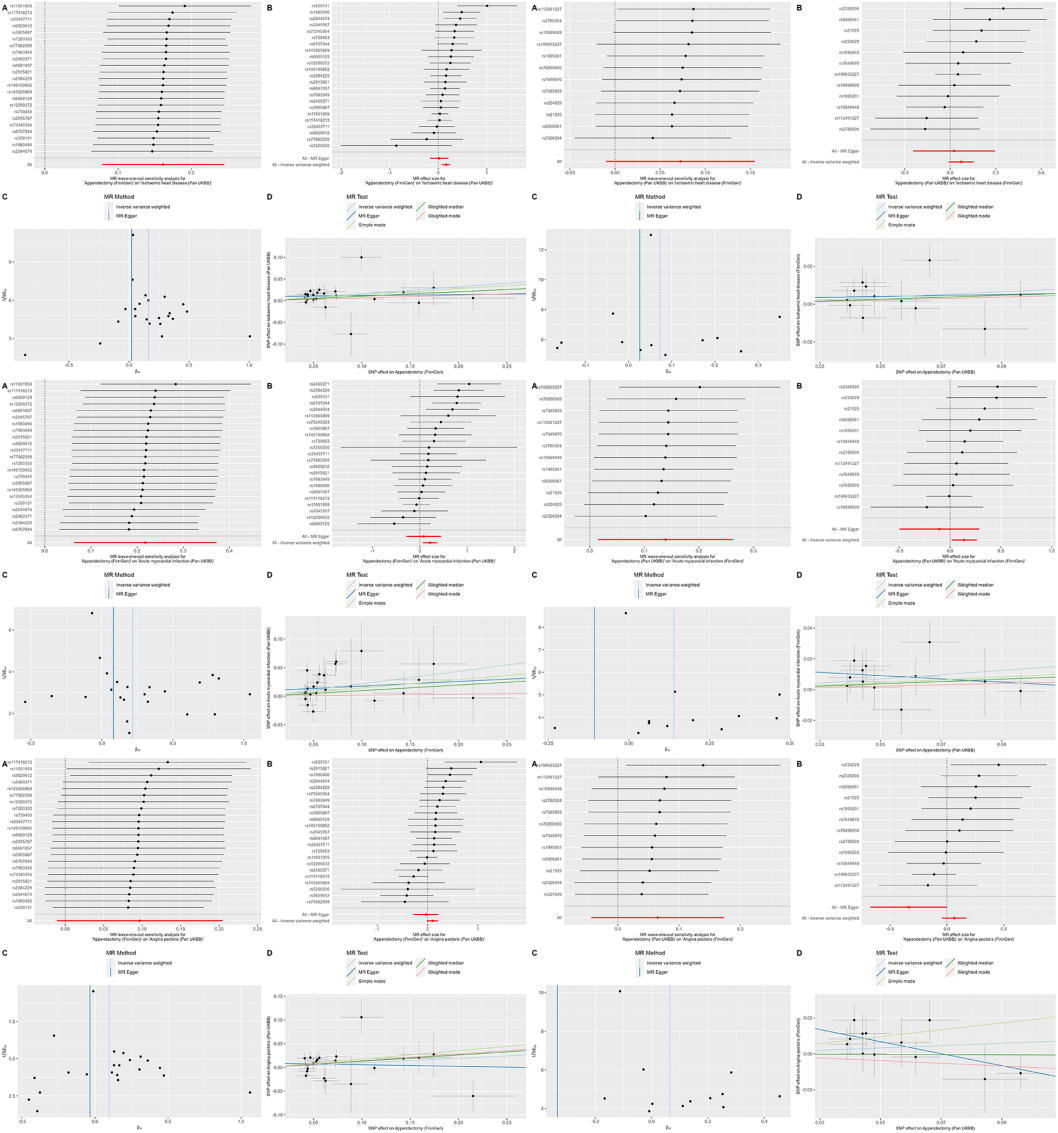
Leave-one-out plots, scatter plots, funnel plots and forest plots for appendectomy on ischaemic heart disease, acute myocardial infarction and angina pectoris.

First, we utilized a discovery cohort to determine the causal relationship between appendectomy and IHD, AMI, and AP (Table 2). The IVW methodology demonstrated a positive correlation between appendectomy and both IHD and its subtypes. That is, IHD (OR: 1.175, 95% CI: 1.089–1.269, *P *= 3.340e-05), AMI (OR: 1.243, 95% CI: 1.098–1.408, *P *= 6.011e-04) and AP (OR: 1.102, 95% CI: 1.008–1.204, *P *= 3.277e-02). These results suggest that appendectomy increases the risk of IHD and its subtypes at the genetic level. Sensitivity analyses showed that the associations between appendectomy and IHD, AMI, and AP were robust and did not show significant heterogeneity or pleiotropy (Table 2). Leave-one-out analyses showed similar results. Scatter plots and funnel plots also demonstrated the stability of the results (Figure 2).

**Table 2:** Causal effects of appendectomy on ischaemic heart disease, acute myocardial infarction and angina pectoris risk in the discovery cohort.

Outcome	Method	SNPs (*n*)	OR (95% CI)	pval	*P*_heterogeneity_	*P*_pleiotropy_
IHD (Pan UKBB)	IVW (fixed effects)	23	1.175 (1.089–1.269)	3.338E-05	2.384E-01	1.261E-01
	MR Egger	23	1.022 (0.845–1.236)	8.259E-01		
	Weighted median	23	1.109 (0.993–1.237)	6.533E-02		
AMI (Pan UKBB)	IVW(fixed effects)	23	1.243 (1.098–1.408)	6.011E-04	5.032E-02	4.306E-01
	MR Egger	23	1.085 (0.752–1.565)	6.675E-01		
	Weighted median	23	1.103 (0.905–1.343)	3.317E-01		
AP (Pan UKBB)	IVW(fixed effects)	23	1.102 (1.008–1.204)	3.277E-02	7.421E-02	2.721E-01
	MR Egger	23	0.966 (0.751–1.243)	7.917E-01		
	Weighted median	23	1.140 (0.996–1.305)	5.785E-02		

SNPs, single nucleotide polymorphisms; IHD, ischaemic heart disease; AMI, acute myocardial infarction; AP, angina pectoris; IVW, inverse variance weighting.

### 3.2 Results of the replication cohort

In the replication cohort, the IVW method showed a positive association between appendectomy and AMI (OR: 1.149, 95% CI: 1.015–1.301, *P *= 2.784e-02), whereas no significant association was found between appendectomy and IHD (OR: 1.075, 95% CI: 0.990–1.168, *P *= 8.372e-02) and AP (OR: 1.067, 95% CI: 0.960–1.186, *P *= 2.284e-01). However, we found significant pleiotropy in the outcomes of appendectomy and AP, and therefore, we could not determine a causal relationship between appendectomy and AP in the replication cohort (Table 3). The results of the leave-one-out method of analysis, scatterplot, and funnel plot are shown in Figure 2.

**Table 3:** Causal effects of appendectomy on ischaemic heart disease, acute myocardial infarction and angina pectoris risk in the replication cohort.

Outcome	Method	SNPs (*n*)	OR (95% CI)	pval	*P*_heterogeneity_	*P*_pleiotropy_
IHD (FinnGen)	IVW (fixed effects)	12	1.075 (0.990–1.168)	8.372E-02	4.564E-01	7.292E-01
	MR Egger	12	1.026 (0.784–1.345)	8.537E-01		
	Weighted median	12	1.053 (0.932–1.190)	4.075E-01		
AMI (FinnGen)	IVW (fixed effects)	12	1.149 (1.015–1.301)	2.784E-02	6.315E-01	2.261E-01
	MR Egger	12	0.899 (0.607–1.332)	6.070E-01		
	Weighted median	12	1.079 (0.911–1.278)	3.802E-01		
AP (FinnGen)	IVW (fixed effects)	12	1.067 (0.960–1.186)	2.284E-01	3.954E-01	3.376E-02
	MR Egger	12	0.716 (0.511–1.001)	7.924E-02		
	Weighted median	12	0.994 (0.859–1.150)	9.325E-01		

SNPs, single nucleotide polymorphisms; IHD, ischaemic heart disease; AMI, acute myocardial infarction; AP, angina pectoris; IVW, inverse variance weighting.

### 3.3 Combined results from the meta-analysis

**Figure 3:**
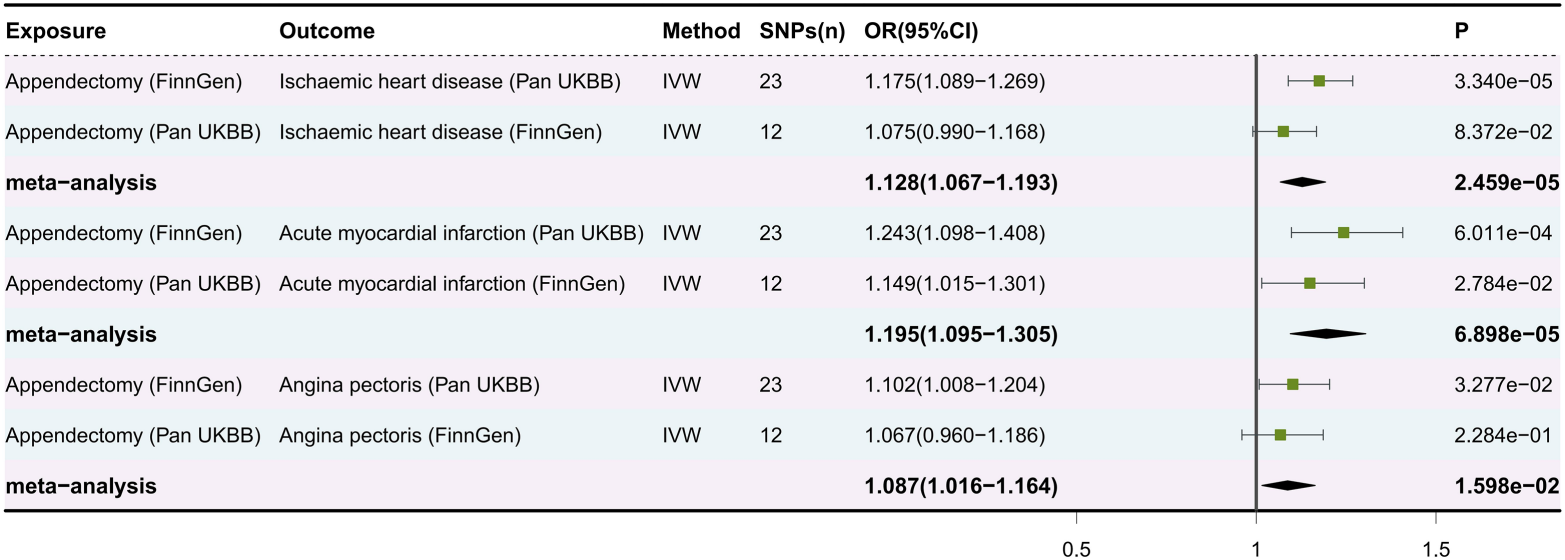
Forest plot of meta-analysis of causal estimation of appendectomy for ischaemic heart disease, acute myocardial infarction and angina pectoris. SNPs, single nucleotide polymorphisms; OR, odds ratio; CI, confidence interval; IVW, inverse variance weighting.

Meta-analysis showed a significant causal relationship between appendectomy and IHD (OR: 1.128, 95% CI: 1.067–1.193, *P *= 2.459e-05), AMI (OR: 1.195, 95% CI: 1.095–1.305, *P *= 6.898e-05) and AP (OR: 1.087, 95% CI: 1.016–1.164, *P *= 1.598e-02) (Figure 3). Unfortunately, even with our rigorous screening, the MR results of appendectomy and angina appeared to be pleiotropic in the validation cohort. Therefore, even though the meta-analysis results suggest that appendectomy is a risk factor for angina, we continue to interpret this result cautiously.

## 4 Discussion

This is the first MR study to systematically assess the causal relationship between appendectomy and IHD, AMI, and AP. Based on the two MR analyses and the final meta-analysis, we found that appendectomy is a significant risk factor for IHD and AMI and a potential risk factor for AP. It provides evidence on screening and testing for IHD in appendectomised patients.

The study by Imre Janszky et al. (10) did not take into account the underlying risk factors for acute myocardial infarction, such as hypertension, hyperlipidemia, and smoking. And the study by Chao-Hung Chen et al. (11) was unable to collect personal information about the participants, such as weight, smoking and dietary habits. These limitations can create uncertainty in the study results. Our comprehensive MR studies, after systematic evaluation, further support their conclusion that appendectomy is a risk factor for IHD and AMI.

No studies explore the causal association between appendectomy and AP. However, our discovery cohort and meta-analysis suggest that appendectomy is a risk factor for AP. Due to significant horizontal pleiotropy in this relationship in the replication cohort, appendectomy may be a potential risk factor for AP. We need more studies to explore this association and the mechanisms involved.

The appendix is a secondary lymphoid organ and an essential component of the mucosa-associated lymphoid tissue system, which is rich in lymphoid tissue and plays a vital role in regulating immunity and intestinal microbiota (10, 29). Removing the appendix and other mucosa-associated lymphoid tissue has immunological effects, such as lowering immunoglobulin A and basophil levels (30). Therefore, it is becoming increasingly important to understand the role of the appendix and the role of appendectomy in disease progression (31). For example, in several animal and human studies, humoral immunity has demonstrated a protective role in atherosclerosis (32). Meanwhile, in addition to finding an association between appendectomy and gastrointestinal disorders, a growing body of research is observing the association between appendectomy and immune disorders such as Hodgkin's lymphoma (33), rheumatoid arthritis (34), and tuberculosis (35). Therefore, paying attention to the impact on the patient's immune system after appendectomy is crucial.

In addition to well-recognized risk factors for coronary artery disease, such as smoking, diabetes, and hypertension, there is growing evidence of the role of inflammation in the development and progression of coronary plaques, as well as their unstable progression and eventual disruption (36, 37). The biological and epidemiological evidence linking inflammation to ischemic heart disease suggests that appendectomy with the removal of mucosa-associated lymphoid tissue may alter atheroprotective immunity. An appendectomy may lead to a decreased capability of the immune system to clear pathogens, resulting in chronic inflammation and an increased risk of IHD (10). Previous studies have shown that the immune system exhibits a protective role in atherosclerosis. For example, humoral immunity is protective against atherosclerosis in several animal and human studies (32).

In addition to appendectomy, Imre Janszky et al. (10) found that tonsillectomy can also increase the risk of AMI. Splenectomy has also been found to be associated with accelerated atherosclerosis (38). These underappreciated secondary lymphoid organs play a unique role in cardiovascular disease. In conclusion, further studies are needed to explore the relationship between appendectomy and cardiovascular diseases such as IHD.

Although the results of our MR study suggest that appendectomy is a risk factor for IHD as well as AMI and AP, this does not mean that we should change our surgical strategy as a result. We place a high priority on postoperative monitoring of appendectomy patients, and we suggest that patients with a history of appendectomy need to be monitored regularly for IHD, especially those with risk factors, which is a critical decision.

Much effort was put into preventing instrumental variables from influencing the study results through confounding factors. We screened SNPs with very stringent criteria. SNPs associated with IHD were excluded by screening through the LDtrait website. e.g., type 2 diabetes mellitus, body mass index-adjusted waist circumference. The above confounders associated with IHD are well recognized, so we excluded them to avoid unreliable results. In addition, we performed MR-PRESSO to remove aberrant SNPs, Cochran's Q-test to detect heterogeneity, and MR-Egger intercept test to detect the presence of horizontal pleiotropy. The stability of our results was also further demonstrated by using the leave-one-out method and other methods. Finally, meta-analysis was used to comprehensively assess the combined causal effect of appendectomy and IHD. The above methods are mainly effective in reducing potential bias and ensuring the reliability of the results.

Our study has several strengths. First, this study is the first MR study to assess the causal relationship between appendectomy and IHD and its subtypes AMI and AP, and the advantage of the MR design in directly detecting causality avoids confounders and reverse causality compared with observational studies. Second, our MR analyses were free of sample overlap and data were obtained from the latest and largest GWAS. Third, we used meta-analyses to comprehensively assess causal effects to ensure the reliability of our results.

However, our study has some limitations. Firstly, we chose a wide threshold in the discovery cohort to obtain sufficient IVs, which may have impacted the results. However, heterogeneity analyses and meta-analyses can ensure the robustness of our results. Secondly, our study could not include more cohorts for meta-analysis due to data limitations. Thirdly, the statistical efficacy of specific analyses is low, which could have made the study results variable, such as the failure to find a causal association between appendectomy and IHD in the replication cohort. Fourthly, our study was conducted mainly in populations of European descent, while the situation in non-European descent still needs to be clarified. Therefore, caution is needed when using our findings in populations of different races and ethnicities. Finally, due to data limitations, we could not conduct further subgroup analyses for variables such as gender, age, and region.

## 5 Conclusion

In conclusion, MR analysis is a reliable method for epidemiological studies, and our findings suggest that appendectomy is a significant risk factor for IHD vs. AMI and a potential risk factor for AP. At the same time, it is crucial to monitor IHD in patients with appendectomy.

## Data Availability

The original contributions presented in the study are included in the article/Supplementary Material, further inquiries can be directed to the corresponding author.
